# Chloroquine efficacy studies confirm drug susceptibility of *Plasmodium vivax* in Chennai, India

**DOI:** 10.1186/1475-2875-13-129

**Published:** 2014-03-31

**Authors:** Sneh Shalini, Saumyadripta Chaudhuri, Patrick L Sutton, Neelima Mishra, Nalini Srivastava, Joseph K David, K John Ravindran, Jane M Carlton, Alex Eapen

**Affiliations:** 1 National Institute of Malaria Research (Indian Council of Medical Research), Sector 8 Dwarka, New Delhi 110 077, India; 2 Department of Biology, Center for Genomics and Systems Biology, New York University, 12 Waverly Place, New York, NY 10003, USA; 3 School of Studies in Biochemistry, Jiwaji University, Gwalior 474 011, India; 4 National Institute of Epidemiology (Indian Council of Medical Research), Second Main Road, Tamil Nadu Housing Board, Ayapakkam, Chennai 600 077, India; 5 National Institute of Malaria Research (Indian Council of Medical Research), National Institute of Epidemiology Campus, Second Main Road, Tamil Nadu Housing Board, Ayapakkam, Chennai 600 077, Tamil Nadu, India

**Keywords:** *Plasmodium vivax*, Chloroquine, *In vitro*, *In vivo*, Genetic diversity, Chennai

## Abstract

**Background:**

Assessing the *Plasmodium vivax* burden in India is complicated by the potential threat of an emerging chloroquine (CQ) resistant parasite population from neighbouring countries in Southeast Asia. Chennai, the capital of Tamil Nadu and an urban setting for *P. vivax* in southern India, was selected as a sentinel site for investigating CQ efficacy and sensitivity in vivax malaria.

**Methods:**

CQ efficacy was evaluated with a 28-day *in vivo* therapeutic study, while CQ sensitivity was measured with an *in vitro* drug susceptibility assay. In both studies, isolates also underwent molecular genotyping to investigate correlations between parasite diversity and drug susceptibility to CQ. Molecular genotyping included sequencing a 604 base pair (bp) fragment of the *P. vivax multidrug resistant gene-1* (*Pvmdr1*) for single nucleotide polymorphisms (SNPs) and also the amplification of eight microsatellite (MS) loci located across the genome on eight different chromosomes.

**Results:**

In the 28-day *in vivo* study (N=125), all subjects were aparasitaemic by Day 14. Passive case surveillance continuing beyond Day 28 in 22 subjects exposed 17 recurrent infections, which ranged from 44 to 148 days post-enrollment. *Pvmdr1* sequencing of these recurrent infections revealed that 93.3% had identical mutant haplotypes (958M/Y976/1076L) to their baseline Day 0 infection. MS genotyping further revealed that nine infection pairs were related with ≥75% haplotype similarity (same allele at six or more loci). To test the impact of this mutation on CQ efficacy, an *in vitro* drug assay (N=68) was performed. No correlation between IC_50_ values and the percentage of ring-stage parasites prior to culture was observed (r_sadj_: -0.00063, p = 0.3307) and the distribution of alleles among the *Pvmdr1* SNPs and MS haplotypes showed no significant associations with IC_50_ values.

**Conclusions:**

*Plasmodium vivax* was found to be susceptible to CQ drug treatment in both the *in vivo* therapeutic drug study and the *in vitro* drug assay. Though the mutant 1076L of *Pvmdr1* was found in a majority of isolates tested, this single mutation did not associate with CQ resistance. MS haplotypes revealed strong heterogeneity in this population, indicating a low probability of reinfection with highly related haplotypes.

## Background

Assessing the *Plasmodium vivax* burden in India is complicated by the potential threat of an emerging chloroquine (CQ) resistant parasite population [1]. *Plasmodium vivax* resistance to CQ is rampant in some regions of Southeast Asia [2,3] and Western Pacific [4], while the emergence of CQ resistance within other regions of the world, including Central and South America [5-7], Southern Asia [8] and even the Indian Subcontinent [9-13] is less evident and represented in the literature mainly in the form of medical case reports. Similar to other countries (reviewed in [14]), CQ resistant *Plasmodium falciparum* emerged in India in the 1970s [15,16]. Decades of research have linked *P. falciparum* drug resistance with a variety of single nucleotide polymorphisms (SNPs) causing non-synonymous amino acid substitutions in the *P. falciparum* CQ resistance transporter gene (*Pfcrt*) [17] and copy number variation in the *P. falciparum* multidrug resistance gene 1 (*Pfmdr1*) [18]. However, mutations within *Pfcrt* appear to be more associated with CQ resistant phenotypes than copy number variation in *Pfmdr1*[19-23]. Comparative studies between *Pfcrt* and the *P. vivax* ortholog, *P. vivax* candidate gene 10 (*Pvcg10*), have not revealed similar functionality [24]. Yet, there is some evidence that specific SNPs causing non-synonymous amino acid substitutions within the *P. vivax* multidrug resistance protein 1 (*Pvmdr1*), the ortholog to *Pfmdr1*, may be associated with drug resistant phenotypes [25].

The *Pvmdr1* gene has been characterized in several global genetic diversity studies in Thailand, Indonesia, Azerbaijan, Turkey, French Guyana, Brazil, Madagascar, Mauritania and most recently in China and India [26-36]. Over a dozen non-synonymous amino acid mutations have been reported [26-35]; of these, Y976F and F1076L are most frequently reported and correlated with CQ resistance [27,34,35,37], although much work remains to irrefutably link these mutations with CQ resistance. In India, a recent study in Kolkata reported an absence of the Y976F mutation in 25 *P. vivax* samples determined to be CQ sensitive taken from patients enrolled in a 28-day *in vivo* efficacy study [29]. Despite the fact that most drug resistant cases remain confined to specific regions in Southeast Asia and the Western Pacific, the impact of long-term CQ exposure on a parasite population is indisputable and has been studied extensively in *P. falciparum.* Recently, Mallick *et al.*[38] showed that a mutant (SVMNT) SNP haplotype of *Pfcrt* was found to predominate during 2002-2006 in regions with high *P. vivax* transmission, in conjunction with high CQ exposure during this time. Due to the fact that CQ remains the first line of defense against *P. vivax*, this highlights the epidemiological impact on the selection of drug resistant parasites in mixed species infections and the maintenance of genetic diversity in a population through inbreeding [38]. Though only a few cases of *P. vivax* CQ resistance have been observed in India [9-13], a selective sweep with a CQ resistant parasite population would have serious consequences for the control of vivax malaria [39-41].

Tamil Nadu, a state located in southern India, is one of several that are heavily burdened by *P. vivax*. The capital city, Chennai, has historically seen high *P. vivax* transmission. For these reasons, Chennai was selected as the sentinel site for investigating CQ sensitivity in *P. vivax*. In this study, the therapeutic efficacy of CQ was evaluated with an *in vivo* study, while CQ sensitivity was measured with an *in vitro* CQ drug assay. In both studies, isolates also underwent molecular genotyping to investigate if a correlation exists between parasite genetic diversity and drug susceptibility to CQ. Molecular genotyping included sequencing a 604 base pair (bp) fragment of *Pvmdr1* for SNP haplotype generation and the amplification of eight microsatellite (MS) loci located across the genome on eight different chromosomes. No detectable CQ resistance was found in the patient samples tested in this study, and there was no evidence of previously correlated *Pvmdr1* resistance alleles gaining in frequency in the parasite population. However, valuable population level genetic diversity information was obtained, and this pilot study paves the way for future studies on the impact of primaquine (PQ) on relapsing infections.

## Methods

### Field location and study design

Samples were collected from patients attending the Central Malaria Laboratory (CML) in George Town, Chennai, in the state of Tamil Nadu, southeast India. George Town, a predominantly residential/commercial area of the city, is densely populated (~51,000 people/km) and mainly comprised of business community members of a high socio-economic status. However, this area also attracts a large number of laborers mainly from the southern districts of Tamil Nadu, and the population is also composed of different ethnic groups from other states of India such as Rajasthan, Bihar, Gujarat, Haryana, Maharashtra, Uttar Pradesh and Nepal. Immigration combined with rapid urbanization within the catchment area of the CML (~2 km or 100,000 residents) maintains local endemicity in this region. The CML is under the administrative control of the Health Department of the Municipal Corporation of Chennai and serves individuals from all economic strata through passive case detection (PCD). Approximately 15,000 blood smear examinations are performed each year.

Samples collected for this study had ethical clearance from the Ethical Committee of the National Institute of Malaria Research (ICMR) and the Institutional Review Board of New York University Langone Medical Center. All participants provided informed consent and/or assent. Inclusion criteria were specific for each study and are detailed below. Patients were treated as per the National Vector Borne Disease Control Programme (NVBDCP) guidelines in India as given in the study methods.

### *In vivo* therapeutic efficacy study

**Figure 1:**
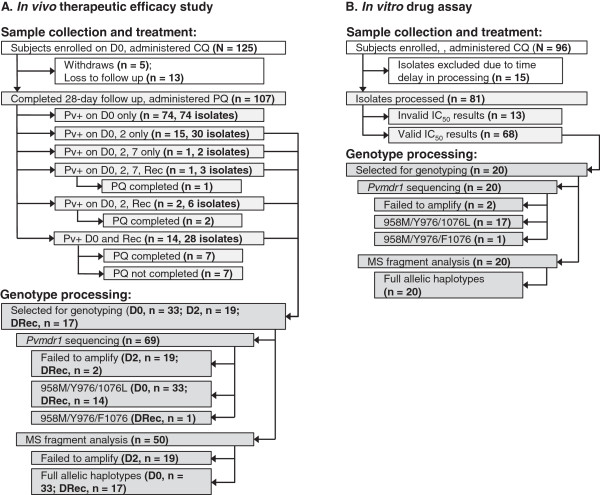
**Study profiles for *in vivo *therapeutic efficacy study (A) and *in vitro *drug assay (B).** Profiles detail enrollment, experimental design and sample utilization for both studies. Abbreviations: CQ (chloroquine), PQ (primaquine), MS (microsatellites), D (day), and Rec (recurrence) and *pvmdr1* (*Plasmodium vivax multidrug resistance gene 1*).

A total of 856 patients were screened for malaria at the CML Malaria Clinic between the start of enrollment in mid-July 2010 and the completion of this study in mid-November 2010. Of these patients, 125 were enrolled in the 28-day *in vivo* therapeutic efficacy study (Figure 1A). Inclusion criteria included: >10 and <70 years of age, non-gravid women, patients with no existing concurrent infection(s) (including mixed species determined by microscopy), parasitaemia >500 asexual parasites/μl blood (to ensure enough parasite DNA for downstream experiments), no administration of anti-malarials or antibiotics within three weeks of enrollment and consent for 28-day follow up. The CML utilizes convenience sampling, which often results in an over-representation of males in the study population; a higher frequency of women and children seek treatment at private clinics. Subjects were enrolled on Day 0 and administered 1,500 mg of CQ over 3 days administered by the CML clinic. Subjects were followed on Days 1, 2, 3, 7, 14, 21, 28 and by passive case surveillance for any clinic visits beyond Day 28 (Day of Recurrence), ranging from 44 to 148 days post-enrollment. PQ (0.25 mg/kg) for radical cure was given on Day 28 and administered for 14 days under direct observation of a medical officer. For each subject, thick and thin smears were collected on Days 0, 2, 7, 14, 21 and 28 and examined by expert microscopists. Blood spots for DNA extraction and isolate genotyping were collected before treatment on Day 0 and on any subsequent day when the subject presented with fever.

To investigate patterns in genetic diversity between infection pairs, DNA was extracted from blood spots and used for (i) amplification and sequencing of a 604 base pair (bp) fragment of *Pvmdr1* to determine the presence of SNPs previously associated with CQ resistance (T958M, Y976F and F1076L) [35] and (ii) MS genotyping to describe the relatedness between the pairs [42], described below.

### *In vitro* CQ drug assay

The *in vitro* drug study, carried out in August 2009 and August-September 2010, enrolled 96 subjects (Figure 1B). Inclusion criteria included: >1 and <70 years of age, non-gravid women, patients with no existing concurrent infection(s) (including mixed species determined by microscopy) and no administration of anti-malarials or antibiotics within 3 weeks of enrollment. Due to the quantity of material collected, no inclusion criterion was established for parasitaemia level. For each subject, thick and thin smears and 3-4 ml of intravenous blood were collected. For each 3-4 ml of patient venous blood, host white blood cells were removed by CF11 filtration within three hours of collection [43], and the packed red blood cells were divided as follows: 1 ml was cryopreserved in Glycerolyte 57 solution (Baxter, Deerfield, IL), 200 μl were spotted onto filter paper and 800 μl was used for the *in vitro* drug susceptibility assay as described [44,45]. Parasite counts taken before and after CF11 filtration revealed a significant reduction in mature blood stage parasites; consequently, no additional synchronization was performed in an effort to maximize immature ring-stage count.

Drug plates were prepared by coating the wells with CQ di-phosphate (Sigma) dissolved in water, serially diluted from a maximum concentration of 514 ng/ml to a minimum of 8 ng/ml, dried in a non-humidified incubator overnight at 37°C, and stored in the dark at 4°C. Batches of plates were tested for efficacy of the drug by using two *P. falciparum* lab strains from the National Institute of Malaria Research (NIMR) Parasite Bank, MRC2 (CQ sensitive) and RKL9 (CQ resistant). Approximately 200 μl of a 2% haematocrit blood medium mixture consisting of McCoy’s 5A media (Gibco) and 20% AB + human serum was added to each well, including control wells, and patient samples were tested in duplicate. Plates were incubated at 37°C in a candle jar for 36 hours, a thick smear prepared for each of the wells, stained with Giemsa or Jaswant-Singh-Bhattacherji (JSB) stain and parasitaemia quantified by microscopy. The number of schizonts per 200 asexual stage parasites was determined for each slide and the result for each drug concentration normalized to the control well. Only healthy schizonts with six or more distinct chromatin dots were quantified. Dose-response data were then analyzed using nonlinear regression analysis (HN-NonLin v.1.1, USAMC-AFRIMS, Bangkok, Thailand), and the 50% inhibitory concentration (IC_50_) obtained.

A selection of isolates used in the *in vitro* drug assay was identified for further characterization by genotyping. Similar to the *in vivo* assay isolates, DNA was extracted from blood spots and used for (i) amplification and sequencing of a 604 base pair (bp) fragment of *Pvmdr1*[35] and (ii) MS genotyping to describe the relatedness between the pairs [42], as described below.

### Genetic diversity analysis

#### *DNA extraction and species-specific PCR*

DNA extraction was performed using QIAamp® DNA Mini Kit (Qiagen Inc., Valencia, CA). Species-specific polymerase chain reaction (PCR) [46] was used to confirm *P. vivax* microscopy diagnosis and rule out mixed species infections.

#### *Amplification and sequencing of Pvmdr1*

Previously published primers and methods were used to amplify a 604 base pair (bp) fragment of *Pvmdr1*[35], capturing three single nucleotide polymorphisms that cause non-synonymous amino acid changes (T958M, Y976F and F1076L) associated with CQ resistance in *P. vivax* in some studies. PCR products were analyzed by gel electrophoresis and sequenced on an ABI 3730*xl* sequencer (Applied Biosystems, Foster City, CA) with ≥ 2x coverage using BigDye Terminator v 3.1 and using both forward and reverse primers. Sequences were viewed using Chromas 2.33 (Technelysium Pvt Ltd), and subsequently aligned using Seaview 4 [47].

#### *Microsatellite amplification and analysis*

Eight MS markers [42] were selected from the literature (Additional file 1) after consideration of recommended guidelines [48]. Using previously published methods and PCR conditions, MS were amplified in 20 μl reactions with ~ 40 ng of extracted DNA using. All reactions were performed individually and not in multiplex. Forward (5′-3′) oligonucleotides were labeled with the phosphoramidite conjugate 6-FAM (Eurofins MWG Operon, Huntsville, AL) and the *P. vivax* laboratory strain Salvador I was used as a positive control. PCR amplicons were analyzed on an ABI 3730*xl* sequencer using GeneScan-500 LIZ size standard (Applied Biosystems, Foster City, CA) for size determination. Bands <200 relative fluorescence units (rfu) were excluded and used to define the background. Isolates with greater than one locus having multiple peaks were defined as multiclonal infections, and minor peaks less than 1/3 the height of the major peak were excluded [49].

Expected heterozygosity (*H*_*e*_) was used to quantify the amount of genetic diversity within each MS locus in this study, but not across compiled haplotypes of loci due to low sample size. *H*_*e*_ was calculated with the following standard formula:

$$H_{e}=\left[\frac{n}{n-1}\right]\left[1-\overset{n}{\underset{i=1}{\sum }}p_{i}^{2}\right],$$

where *n* is the number of infections sampled and *p*_*i*_ is the frequency of the *i*^th^ allele. For the maximum expected probability of allelic combinations ranging from one to eight loci, let *P* equal the maximum probability of *A*, highest frequency alleles, to the *n*^*th*^ locus, defined as:

$$Ρ\left({Α}_{1}\cap {Α}_{2}\cdots \cap {Α}_{n}\right)=Ρ\left({Α}_{1}\right)Ρ\left({Α}_{2}\right)\cdots Ρ\left({Α}_{n)}\right).$$

Due to the small sample size, it is not possible to accurately estimate the allele frequencies for the population. To account for this potential error, the standard deviation (SD) of the allele frequency was estimated by a parametric bootstrap procedure, which generates random samples from a population assumed to have allele frequencies equal to their maximum-likelihood values. The SD of each allele frequency was then estimated from the resulting distribution of allele frequencies [50,51]. The resulting SD was used to calculate the standard error (SE) by: SE=SD√n , where *n* is equal to the number of samples with a specific allele.

## Results

### *In vivo* therapeutic efficacy study

**Figure 2:**
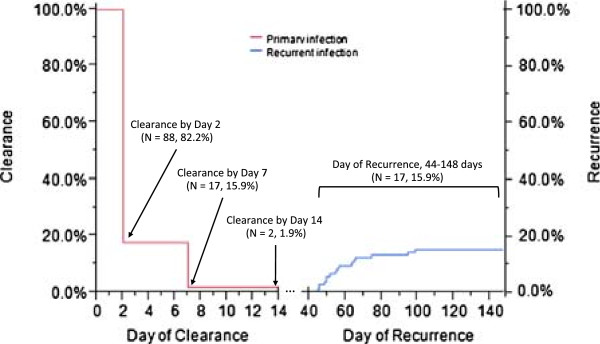
**Clearance and recurrence plot of the infections observed in the *in vivo *therapeutic efficacy study.** Of the 125 subjects enrolled in this study 107 completed the 28-day follow up. An absence of fever within 48 hours (Day 2) was observed for all 107 subjects completing the study. Eighty-eight (82.2%) subjects were aparasitaemic for both sexual and asexual morphological stages by Day 2 and 17 (15.9%) by Day 7 (Primary infection, red line). Two (1.9%) subjects remained positive for sexual stage parasites until a time between Day 7 and Day 14, at which point no parasites remained detectable by microscopy (Primary infection, red line). Of these 107 subjects, 17 subjects were parasite positive at a time point beyond the 28-day enrollment period, ranging from 44-148 days post-enrollment (Recurrent infection, blue line).

To determine the *in vivo* therapeutic efficacy of CQ, 125 patients were enrolled in a 28-day follow up study at the CML in George Town, Chennai. Demographic and clinical data is provided in Table 1A. A total of 107 (85.6%) subjects completed the 28-day follow up, five subjects (4.0%) withdrew from the study and 13 (10.4%) were lost to follow up (Figure 1A). No fever was observed after 48 hours (Day 2) for all 107 subjects completing the study. Eighty-eight (82.2%) subjects had no sexual or asexual parasites by Day 2, and 17 subjects (15.9%) had no sexual or asexual parasites by Day 7. Two (1.9%) subjects remained positive for sexual stage parasites until a time between Day 7 and Day 14, at which point all patients remained negative for *P. vivax* for the remainder of the 28-day study (Figure 2). Though the study was completed on Day 28, passive case surveillance continued to monitor the enrolled subjects until four months post-enrollment. Twenty-two of the subjects who had completed the 28-day follow up presented with malaria-like symptoms at a time-point after Day 28. These 22 subjects were tested for malaria by microscopy and 17 subjects were found to have recurrent *P. vivax* infections on a day ranging from 44 to 148 post-enrollment (termed “Day of Recurrence”) (Figure 1A).

**Table 1:** **Demographic and clinical data for the *in vivo *** **therapeutic efficacy study (A) and the *in vitro *** **drug assay (B)**

**A. *In vivo *** **therapeutic efficacy study**			**B. *In vitro *** **drug assay**		
**Characteristics**		**(N = 125)**	**Characteristics**		**(N = 96)**
**Sex [n (%)]**	Male	120 (95.0)	**Sex [n (%)]**	Male	85 (88.5)
	Female	5 (5.0)		Female	11 (11.5)
**Wt (kg)**			**Age (yr)**		
	Mean	52.8		Mean	23.9
	Range	5.0-77.0		Range	7-53
	SD	±10.4		SD	8.3
	95% CI	51.0-54.7		95% CI	22.2-25.6
**Age (yr)**			**Parasite count (n/μl)**		
	Mean	25.9		Mean	6348.6
	Range	13-66		Range	480-21,522
	SD	10.5		SD	4390.8
	95% CI	24.0-27.8		95% CI	5,172.8-7,524.5
**Body temperature (°F)**			**Parasitaemia (%)**		
	Mean	98.5		Mean	0.163
	Range	95.4-104.4		Range	0.032-0.445
	SD	2.2		SD	0.094
	95% CI	98.1-98.9		95% CI	0.122-0.203
**Haemoglobin (g/dl)**			**Rings (%)**		
	Mean	14.4		Mean	3.2
	Range	12.0-16.6		Range	0.0-33.3
	SD	1.0		SD	5.9
	95% CI	14.2-14.6		95% CI	1.9-4.6
**Parasite count (n/μl)**			**Trophozoites (%)**		
	Mean	9049.2		Mean	91.0
	Range	1,000-57,200		Range	58.3-100.0
	SD	6697.3		SD	11.6
	95% CI	5,243.4-7,624.4		95% CI	88.4-93.6
			**Schizonts (%)**		
				Mean	5.8
				Range	0.0-33.3
				SD	1.1
				95% CI	3.6-7.9

### Genetic diversity and *Pvmdr1* genotyping of *in vivo* isolates

Day 0 infections that were subsequently positive on Day 2 and/or Day of Recurrence were genotyped. Despite detecting two positive cases on Day 7, no blood was collected due to an absence of fever (see Methods), so these infections were excluded from genotyping experiments. In total, 69 isolates (33 Day 0, 19 Day 2 and 17 Day of Recurrence) were selected for genotyping in the following combinations: (1) Day 0 and 2 paired-infection (n = 15, 30 isolates); (2) Day 0, 2 and 7 paired-infection (n = 1, 2 isolates); (3) Day 0, 2, 7 and Day of Recurrence paired-infection (n = 1, 3 isolates); (4) Day 0, 2 and Day of Recurrence (n = 3, 6 isolates); and (5) 14 Day 0 and Day of Recurrence paired-infection (n = 14, 28 isolates) (Figure 1A). No Day 2 isolates successfully amplified for genotyping or sequencing, despite successful amplification by species-specific diagnostic PCR. Consequently, the only paired-infection suitable for genetic analysis were Day 0 (n = 33) and Day of Recurrence (n = 17) (Figure 1A).

A 604bp fragment of *Pvmdr1* was successfully amplified and sequenced for all Day 0 isolates (n = 33); all isolates (100%) were identified as having the mutant 958M/Y976/1076L haplotype. For the Day of Recurrence isolates, 15 of the 17 isolates successfully amplified and were sequenced; 14 (93.3%) of these were identified as the mutant 958M/Y976/1076L haplotype and 1 (6.7%) as the mutant 958M/Y976/F1076 haplotype (Table 2). No new SNPs were detected and sequences have been deposited in GenBank (Accession nos: KC818367-KC818412).

**Table 2:** **Genotyping results from isolates in the *in vivo *** **therapeutic efficacy study and *in vitro *** **drug assay**

**Study**		**Subjects enrolled (N)**	***Pvmdr1 *** **haplotypes**^**a**^			**Microsatellite genotyping**			
			**Isolates successfully genotyped (n)**	**958M/Y976/1076L (n (%))**	**958M/Y976/F1076 (n (%))**	**Isolates successfully genotyped (n)**	**Multiclonal infections (n (%))**	**Clones (n)**	**Multiplicity of infection**^**c**^
***in vivo *** **therapeutic efficacy:**									
	Day 0	125	33	33 (100)	0 (0.0)	33	5 (15.2)	38	1.15
	Day 3	19	0	-	-	0	-	-	-
	Day Other	17	15	14 (93.3)	1 (6.7)	17	3^b^ (17.6)	21	1.24
	**Total**	**125**	**48**	**47 (97.9)**	**1 (2.1)**	**50**	**8 (16.0)**	**59**	**1.18**
***in vitro *** **drug assay:**									
	Group 1	51	9	8 (88.9)	1 (11.1)	10	4 (40.0)	14	1.40
	Group 2	17	9	9 (100)	0 (0.0)	10	2 (20.0)	12	1.20
	**Total**	**68**	**18**	**17 (94.4)**	**1 (5.6)**	**20**	**6 (30.0)**	**26**	**1.30**

^a^GenBank accession nos: KC818349-KC818412; *Pvmdr1* haplotype of the CQ sensitive Salvador I reference strain is T958/Y976/F1076.

^b^Of the three multiclonal infections, two had two alleles at more than one locus (double infection) and one had three alleles at more than one locus (triple infection).

^c^Multiplicity of infection is the total number of clones detected divided by the total number of infections.

Eight MS markers were used to determine the genetic diversity of the Day 0 (n = 33) and Day of Recurrence (n = 17) isolates (Figure 1A). MS haplotypes were found to be extremely diverse with no common haplotypes within a single group (within the Day 0 samples or within the Day of Recurrence samples). However, when comparing paired isolates collected from the same individual (on Day 0 paired with Day of Recurrence), there were distinct patterns of relatedness, likely indicating relapsing or recrudescing parasite infections. Of the 17 subjects that were parasite positive on Day of Recurrence, MS haplotypes revealed that 58.8% (n = 9) of the infection pairs were related to the Day 0 sample with ≥75% haplotype similarity (same allele at six or more loci) between samples. In fact, there were six instances where all eight (100%) MS alleles matched exactly, one instance where seven (87.5%) alleles matched and two instances with six (75.0%) alleles matched (Table 3). The probability of detecting identical allelic combinations was calculated for each paired sample based on the number of alleles repeated and the observed frequency of each specific allele present within the population (see Methods for calculation parameters and equation). Due to the extensive genetic diversity within the local *P. vivax* parasite population, it seems unlikely that any Day of Recurrence infection identified as having ≥75% relatedness between sample pairs was due to a new infection, with a probability ranging from 0.001 to 1.98E-05; rather it is more likely to be the result of a relapsing or recrudescing parasite infection.

**Table 3:** Microsatellite allele sharing between paired-infections, and the calculated maximum expected probability of sharing a specific combination of alleles

**Possible alleles shared between pairs (n/total)**	**Infection(s) with allele pair profile (n/total)**	**Maximum expected probability (SE)**	**Subject completing PQ treatment (n/total)**
1/8	4/17	0.422 (0.410, 0.434)	2/4
2/8	1/17	0.119 (0.11, 0.128)	0/1
3/8	1/17	0.032 (0.028, 0.036)	0/1
4/8	1/17	0.008 (0.007, 0.010)	1/1
5/8	1/17	0.002 (0.001, 0.003)	1/1
6/8	2/17	5.23E-04 (3.90E-04, 6.92E-04)	1/2
7/8	1/17	1.23E-04 (8.60E-04, 1.72E-04)	1/1
8/8	6/17	2.68E-05 (1.76E-05, 4.00E-05)	4/6

Although all 17 subjects with recurrent infections began the 14-day PQ drug regimen during their enrollment in the 28-day *in vivo* study, only 10 were known to complete the recommended therapy. MS data from this study indicated that six of the nine subjects with highly related paired-infections did complete radical PQ therapy. This finding may support recurrence via recrudescence if the parasite population remains susceptible to the drug, or recurrence via relapse if the parasite population has become tolerant or resistant (Table 3).

Of these 17 paired-infections, 13 (76.5%) were single infections on both Day 0 and Day of Recurrence, while two (11.8%) were single on Day 0 and then multiclonal on Day of Recurrence, one (5.9%) was multiclonal on Day 0 and then single on Day of Recurrence, and one (5.9%) was multiclonal on both Day 0 and Day of Recurrence. In all four cases, all possible haplotype combinations were made and compared between paired-infections. Given the high allelic diversity, only two of the four multiclonal infections shared ≥75% haplotype similarity, with both cases sharing six of eight alleles (Table 3).

### *In vitro* CQ drug assay

A total of 96 subjects were enrolled by PCD at the CML to assay for susceptibility to CQ *in vitro*. Demographic and clinical data is provided in Table 1B. An *in vitro* CQ drug assay completed on 81 isolates gave reliable IC_50_ values for 68 isolates (Figure 1B). IC_50_ values were low for all isolates, ranging from 7.8-30.2 nM. When plotted the distribution of the IC_50_ values is a non-linear curve, with a geometric mean of 16.3 nM (95% CI: 14.9-17.8 nM).

Unlike reports from other vivax malaria endemic regions, the percentage of ring-stage parasites upon collection and processing was low. The mean parasitaemia was 0.163% (95% CI: 0.122-0.203%) and was largely dominated by trophozoite-stage parasites (90.0%, 95% CI: 88.4-93.6%). This resulted in a reduced ring-stage inoculation of *in vitro* culture (Mean: 3.23%, 95% CI: 1.90-4.58%). After 36 hours of *in vitro* culture, no correlation between IC_50_ values and the percentage of ring-stage parasites prior to culture was observed (r_sadj_: -0.00063, p = 0.3307).

### Genetic diversity and *Pvmdr1* genotype of *in vitro* samples

The interquartile range of the IC_50_ values was used to group isolates into either Group 1 (n = 40, IQR <75% = 7.8 to 21.9) or Group 2 (n = 18, IQR ≥75% = 22.1 to 30.2), and a total of 20 isolates (10 from each group) were selected for further analysis (Figure 1B). Genotyping isolates across a range of IC_50_ values helped to gather evidence of *Pvmdr1* resistance alleles gaining in frequency in the parasite population.

*Pvmdr1* was successfully amplified and sequenced for nine of the 10 isolates in Group 1; eight of these (88.9%) were identified as having the mutant 958M/Y976/1076L haplotype and 1 (11.1%) as having the mutant 958M/Y976/F1076 haplotype. A similarly dominant pattern of the mutant 958M/Y976/1076L haplotype was observed in nine of the Group 2 isolates (100%). These sequences have been deposited in GenBank (Accession nos: KC818349-KC818366).

The same 20 isolates were genotyped using eight polymorphic MS to obtain background genetic diversity data in the form of the mean number of alleles per locus, the distribution of alleles per locus and the multiplicity of infection (MOI) (Table 2). Both groups had high levels of genetic diversity with no common MS haplotypes within or between the two groups. Though expected given the small sample size, there was no significant difference between the mean number of alleles per locus for Group 1 isolates was 5.89 (range = 4 to 9, SD = 1.55) and for Group 2 isolates 6.5 (range = 3 to 8, SD = 1.93). The distribution of specific alleles between the two groups also did not differ significantly (ranging from r^2^ = 0.17 and p = 0.18, to r^2^ = 0.06 and p = 0.60); that is, there did not appear to be a distribution of alleles that was specific to either group. Similarly, there was no significant difference between the total number of clones, multiclonal infections or MOI between Group 1 (n = ≥14, n = 4 and MOI = 1.40, respectively) and Group 2 (n = ≥12, n = 2 and MOI = 1.20, respectively) (Table 2).

### A city-wide view of *P. vivax* genetic diversity in Chennai

Since the subjects for the *in vivo* and *in vitro* studies were collected from the same clinic, with sample collection occurring during the same period (2009-2010), MS data were collated into one database to provide a more comprehensive perspective of the parasite population-level diversity. In total 70 samples were genotyped, 20 from the *in vitro* CQ drug assay and 50 samples from the *in vivo* therapeutic efficacy study. For each locus the number of alleles, size range, frequency of the most common allele, expected heterozygosity (*H*_*e*_) and the MOI was calculated (Table 4). On the whole, the mean number of alleles per locus was high (Mean = 13.3, SD = 4.71, range = 5 to 20), indicating high levels of *P. vivax* genetic diversity, which was confirmed by calculating *H*_*e*_ (Mean = 0.856, SD = 0.063, range = 0.729 to 0.912). As expected, the number of alleles per locus was positively correlated with the *H*_*e*_ (p = 0.0008, r^2^ = 0.86, ANOVA) and negatively correlated with the frequency of the most common allele per locus (p = 0.0029, r^2^ = 0.79, ANOVA). Consistent with this order, the frequency of the most common allele per locus was also negatively correlated with the *H*_*e*_ (p = 0.0002, r^2^ = 0.92, ANOVA) and the MOI (p = 0.0206, r^2^ = 0.62, ANOVA). MOI was only significantly correlated with the frequency of the most common alleles for each locus, indicating that it is not necessarily the amount of diversity present within a high transmission population, but rather how the diversity is dispersed within the population that influences the accumulation of multiple clones within a single infection.

**Table 4:** **Genetic diversity of eight *P. vivax *** **microsatellite loci in the study population**

**Locus**	**No. of alleles**	**Size range**	**Frequency of most common allele (SD)**	**Multiplicity of infection**	***H***_***e***_
**MS2**	10	188-250	0.281 (0.057)	1.15	0.807
**MS3**	10	169-199	0.250 (0.055)	1.13	0.852
**MS6**	15	197-269	0.250 (0.055)	1.09	0.882
**MS7**	5	131-143	0.422 (0.062)	1.01	0.732
**MS9**	15	137-197	0.226 (0.056)	1.18	0.860
**MS10**	17	159-250	0.219 (0.052)	1.13	0.906
**MS12**	14	183-311	0.266 (0.056)	1.10	0.862
**MS20**	20	160-223	0.234 (0.053)	1.14	0.882

## Conclusions

In this study, *P. vivax* CQ sensitivity was investigated among infected patients in Chennai, Tamil Nadu. A longitudinal *in vivo* efficacy study was used to monitor the clearance of parasites and recurrence of infection, and an *in vitro* CQ drug assay was used to monitor the dose-response effect on parasites isolated from patient samples (Table 1 and Figure 1). Molecular genotyping was used in both studies to investigate the genetic structure of the parasites and to determine if a correlation between parasite genetic diversity and drug susceptibility to CQ existed.

The *in vivo* efficacy study demonstrated *P. vivax* sensitivity to CQ in Chennai. Of the 125 subjects enrolled, 107 completed follow up and were microscopy negative for asexual parasites by Day 7; however, two subjects remained positive for sexual parasites until a time prior to the Day 14 collection. Seventeen subjects remained microscopy negative until a time point after Day 28, called Day of Recurrence, which ranged from 44 to 148 days after the initial enrollment (Day 0) (Figure 2). Isolates from Day 0 and Day of Recurrence were genotyped to determine relatedness between the infecting clones. Of the 17 paired-infections, nine were found to share ≥75% of the same alleles. Six (66.7%) of the nine subjects experiencing recurrent infections were known to take PQ radical treatment beginning on Day 28 of this study (Table 3). Though no distinction can be made on whether or not these recurrent infections were due to a relapse, recrudescence or reinfection, microscopy examination indicated parasite clearance by Day 7 and the time-to-recurrence for these infections was within the expected timeframe of a tropical relapse [52,53]. Other studies in India have reported variable *P. vivax* relapse rates, between 2.2 and 40.1%, which may be linked with the geographic distribution of relapsing phenotypes [54-57]. However, as recently highlighted by White (2011), little is known about these relapsing phenotypes in India, which is a serious cause for concern given that India harbors a significant global *P. vivax* burden [58,59]. In an effort to understand these phenotypes, cross-sectional, longitudinal and clinic epidemiological studies are underway at three sentinel sites in India, as part of the Center for the Study of Complex Malaria in India (CSCMi), an International Center of Excellence in Malaria Research [60].

Results from the *in vitro* CQ drug assay, which measures the inhibitory concentration (IC_50_) of drug needed to prevent parasite growth, also revealed *P. vivax* sensitivity to CQ in the parasite isolates collected from Chennai. IC_50_ values from this study ranged from 7.8-30.2 nM (N = 68), which is significantly lower than the 220 nM cutoff for CQ resistance define by Suwanarusk *et al.*[35] in Indonesia and Thailand, as well as the 100 nM cutoff for CQ resistance defined by Druilhe *et al*. [61] in isolates from Myanmar. Regional differences in the above cutoff values are most likely due to methodological differences. Fragment sequencing of *Pvmdr1* in both studies revealed that the mutant 958M/Y976/1076L was the dominant haplotype, while the mutant 958M/Y976/F1076 was the minor haplotype (Table 2). The majority of isolates containing the 1076L mutation, which is highly prevalent across the globe, is actually predominantly found in regions with *P. vivax* CQ-sensitivity [26-28,35,37,62,63]. The detection of these two haplotypes is consistent with a recent report in nearby Nepal, which may indicate relatedness between the parasite populations in India and Nepal [63]. Similar to a genotyping study in Kolkata [29], the mutant 976F was not detected in any of the Chennai isolates tested in these two studies (Table 2). Previous studies have reported that this mutation is linked with drug resistance [35,63]; however, subsequent studies have been unable to confirm this association [64-66]. MS genotyping of these *P. vivax* isolates revealed an unconstrained and highly diverse population of parasites, with no correlation to *Pvmdr1* haplotypes or IC_50_ values. Rather, valuable information was gained about the population-level genetic diversity of extant *P. vivax*, which will help to direct future population diversity studies with larger sample sizes.

Considering the highly endemic nature of *P. vivax* in India and proximity to other countries such as Myanmar, Thailand and Indonesia, the emergence of *P. vivax* CQ resistance is a persistent threat. Although there are few case reports of *P. vivax* resistance to CQ in India, the potential for a similar devastating situation as happened with the emergence of CQ resistant *P. vivax* in Southeast Asia should encourage constant surveillance. Urban malaria epicenters, like Chennai, are of particular interest because the local incidence is not only shaped by common environmental factors, such as the seasonality of monsoons and the development of insecticide resistance, but also by population density, rapid urbanization, sanitation practices and inaccessibility to many sites for continuous monitoring. Though it is not completely understood why *P. vivax* drug resistance has yet to sweep across India, further investigation on this subject is currently underway as part of CSCMi. As India moves into the pre-elimination phase, the need for increased surveillance of drug resistance has never been more important.

## Competing interests

The authors declare no competing interests.

## Authors’ contributions

AE and JMC designed the study protocol and provided training for the study; SS, SC undertook sample collection, *in vitro* drug tests and analysis with help from JC and AE. PLS, SC and SS performed molecular biology experiments; PLS undertook data and statistical analysis; AE, JKD and KJR organized logistics for students, provided access to laboratory space and equipment and provide expertise to facilitate the study protocol; NM and NS provided mentoring for SS; PLS, SS, SC, JC and AE wrote the manuscript. All authors read and approved the final manuscript.

## Authors’ information

Sneh Shalini, Saumyadripta Chaudhuri and Patrick L Sutton joint first author.

## Supplementary Material

Additional file 1 **Primer information for microsatellites used for genotyping *Plasmodium vivax* parasites.** Click here for file
