# Supplementary material for: Chloroquine efficacy studies confirm drug susceptibility of Plasmodium vivax in Chennai, India
Source: Malar J. 2014 Mar 31;13:129. doi: 10.1186/1475-2875-13-129 (PMC4021252; doi:10.1186/1475-2875-13-129)
Supplement: Additional file 1 — Primer information for microsatellites used for genotyping Plasmodium vivax parasites. [file 1475-2875-13-129-S1.docx]

**Additional File 1. Primer information for microsatellites used for genotyping *Plasmodium vivax* parasites.**

| **Microsatellite loci** | **Chromosome no.** | **Motif** | **No. study sites tested^1-7^** | **Size ranges detected^1-7^** |
| --- | --- | --- | --- | --- |
| **MS2** | 6 | (TAAA)_2_TATA(TAAA)_6_TATA(TAAA)_19_ | 11 | 170-315 |
| **MS3** | 4 | (GAA)_11_ | 13 | 152-200 |
| **MS6** | 11 | (TCC)_2_(TCT)_3_(CCT)_2_(TCC)_2_GCTTCT(TCC)_10_ | 10 | 157-260 |
| **MS7** | 12 | (GAA)_9_ | 11 | 124-212 |
| **MS9** | 8 | (GGA)_18_ | 11 | 139-182 |
| **MS10** | 13 | GAA(GGA)_2_AGA(GGA)_9_AGA(GGA)_4_AGAGGAAGA (GGA)_3_AGAGGAAGA(GGAAAA)_4_(GGA)_2_(AGA)_11_ (GGA)_3_AGA)_2_GGAAGA(GGA)_2_ | 13 | 174-306 |
| **MS12** | 5 | (TTC)_10_(TGC)_4_ | 11 | 191–263 |
| **MS20** | 10 | (GAA)_11_GAG(GAA)_13_(CAA)_4_GAA(CAA)_5_ | 13 | 150–246 |

1. Ferreira MU, Karunaweera ND, da Silva-Nunes M, da Silva NS, Wirth DF, Hartl DL: **Population structure and transmission dynamics of *Plasmodium vivax* in rural Amazonia.** *J Infect Dis* 2007, 195:1218-1226.

2. Gunawardena S, Karunaweera ND, Ferreira MU, Phone-Kyaw M, Pollack RJ, Alifrangis M, Rajakaruna RS, Konradsen F, Amerasinghe PH, Schousboe ML, Galappaththy GN, Abeyasinghe RR, Hartl DL, Wirth DF: **Geographic structure of *Plasmodium vivax*: microsatellite analysis of parasite populations from Sri Lanka, Myanmar, and Ethiopia.** *Am J Trop Med Hyg* 2010, 82:235-242.

3. Honma H, Kim JY, Palacpac NM, Mita T, Lee W, Horii T, Tanabe K: **Recent increase of genetic diversity in *Plasmodium vivax* population in the Republic of Korea.** *Malar J* 2011, 10:257.

4. Orjuela-Sánchez P, da Silva NS, da Silva-Nunes M, Ferreira MU: **Recurrent parasitemias and population dynamics of *Plasmodium vivax* polymorphisms in rural Amazonia.** *Parasitology* 2009, 136:1097-1105.

5. Van den Eede P, Erhart A, Van der Auwera G, Van Overmeir C, Thang ND, Hung le X, Anne J, D'Alessandro U: **High complexity of *Plasmodium vivax* infections in symptomatic patients from a rural community in central Vietnam detected by microsatellite genotyping.** *Am J Trop Med Hyg* 2009, 81:961-968.

6. Van den Eede P, Van der Auwera G, Delgado C, Huyse T, Soto-Calle VE, Gamboa D, Grande T, Rodriguez H, Llanos A, Anné J, Erhart A, D'Alessandro U: **Multilocus genotyping reveals high heterogeneity and strong local population structure of the *Plasmodium vivax* population in the Peruvian Amazon.** *Malar J* 2010, 9:151.

7. Zeyrek FY, Tachibana S, Yuksel F, Doni N, Palacpac N, Arisue N, Horii T, Coban C, Tanabe K: **Limited polymorphism of the *Plasmodium vivax* merozoite surface protein 1 gene in isolates from Turkey.** *Am J Trop Med Hyg*, 83:1230-1237.
